# Gallbladder Volvulus Presenting as Acute Appendicitis

**DOI:** 10.7759/cureus.14484

**Published:** 2021-04-14

**Authors:** Neal Shah, Eric Ballecer, Iman Hanna, Galina Levin, Michael E Khalife

**Affiliations:** 1 Department of Internal Medicine, New York University (NYU) Langone Hospital—Long Island, Mineola, USA; 2 Department of Gastroenterology and Hepatology, New York University (NYU) Langone Hospital—Long Island, Mineola, USA; 3 Department of Pathology, New York University (NYU) Langone Hospital—Long Island, Mineola, USA; 4 Department of Radiology, Memorial Sloan Kettering Nassau, Uniondale, USA; 5 Department of General Surgery, New York University (NYU) Langone Hospital—Long Island, Mineola, USA

**Keywords:** gallbladder volvulus, necrosis, biliary

## Abstract

Gallbladder volvulus is a rarely reported and diagnosed condition. We present a case of an elderly female with right lower quadrant pain mimicking acute appendicitis without conclusive imaging; however, due to worsening serological laboratory findings and sepsis picture, an exploratory laparotomy was performed. A necrotic gallbladder was removed, diagnosing gallbladder volvulus. A systemic literature review showed the difficulty in making a diagnosis and the uniqueness of our patient presentation. A high level of clinical suspicion for gallbladder volvulus must be maintained and should be included in the differential diagnosis in elderly women with an acute abdomen, as complications can be severe.

## Introduction

Gallbladder volvulus (GV) occurs when the organ twists along its long axis, resulting in vascular compromise. This condition affects women more often and has a higher incidence in the geriatric population. There have been only 400 cases recorded since it was first described in 1898; however, its incidence appears to have increased in the twenty-first century as the average life expectancy increases [1]. The presenting symptoms of GV can include an abdominal mass, pain, with or without nausea and vomiting, and can mimic many other disease processes.

We present a case of an elderly female with GV who experienced acute lower abdominal pain in the setting of a palpable abdominal mass.

This article was previously presented as a meeting abstract at the ACG 2020 Annual Scientific Meeting & Postgraduate Course on October 23, 2020.

## Case presentation

**Figure 1:**
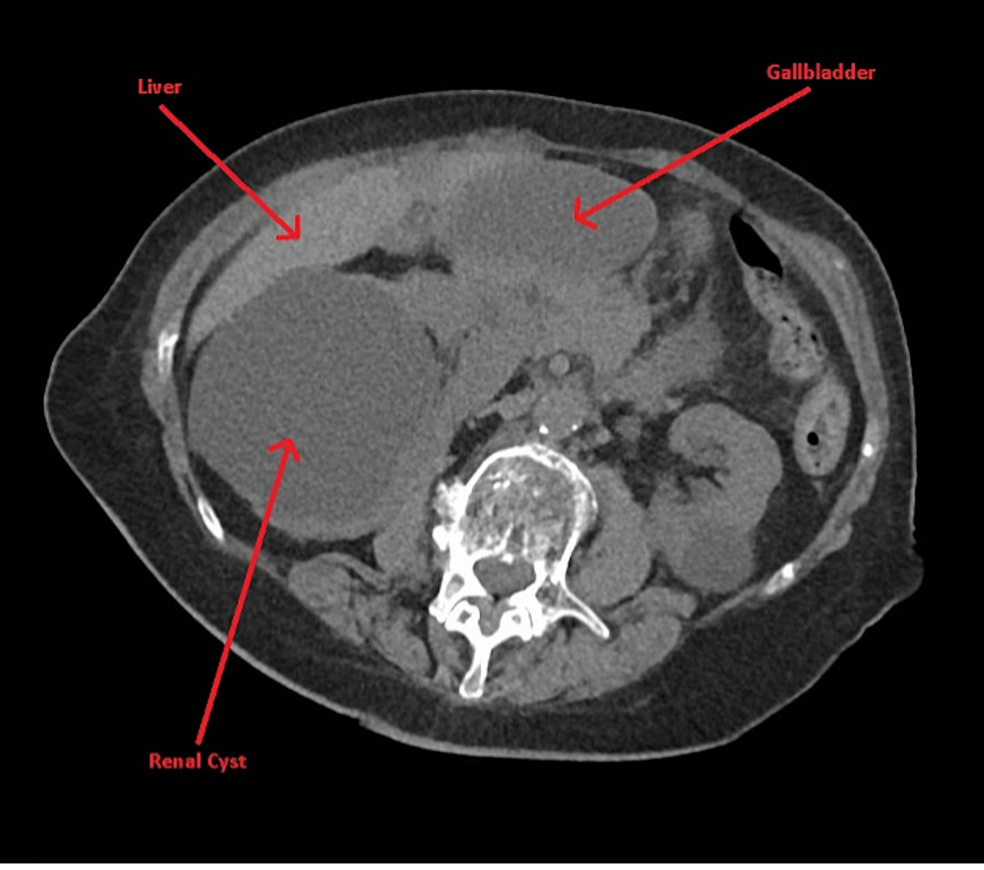
CT Abdomen and Pelvis Axial CT image of the abdomen performed without intravenous contrast demonstrates a distended, horizontally positioned gallbladder. The actual twist in the gallbladder neck is not well seen. There is no pericholecystic inflammation and no appreciable gallbladder wall thickening.

**Figure 2:**
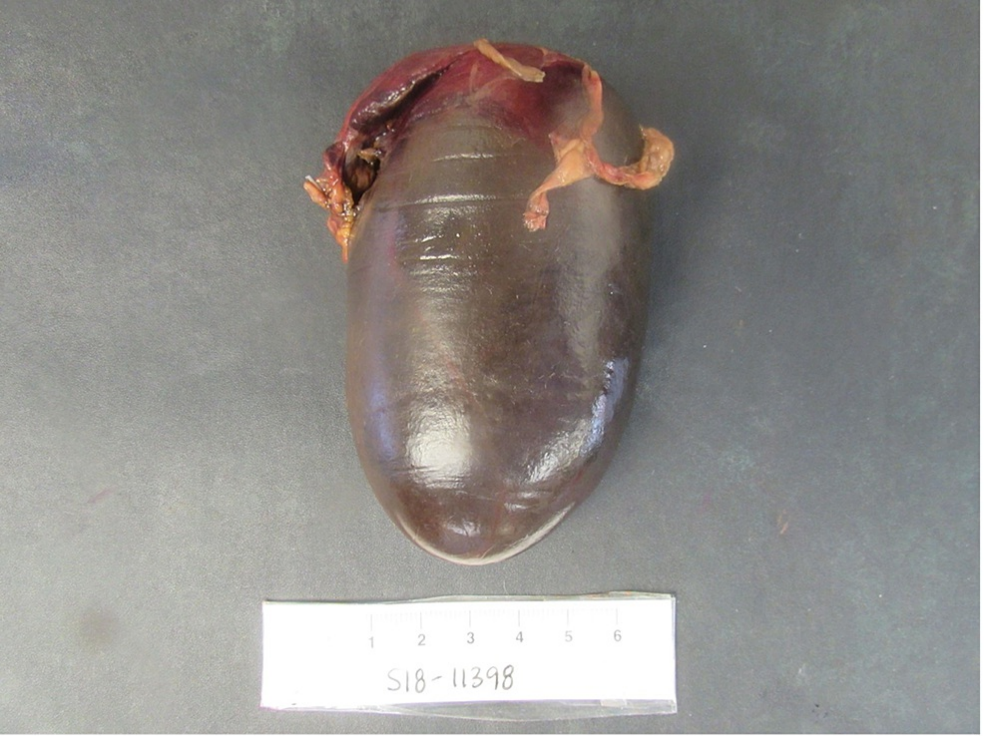
Gallbladder Grossly the gallbladder is enlarged, distended, necrosed, and congested.

**Figure 3:**
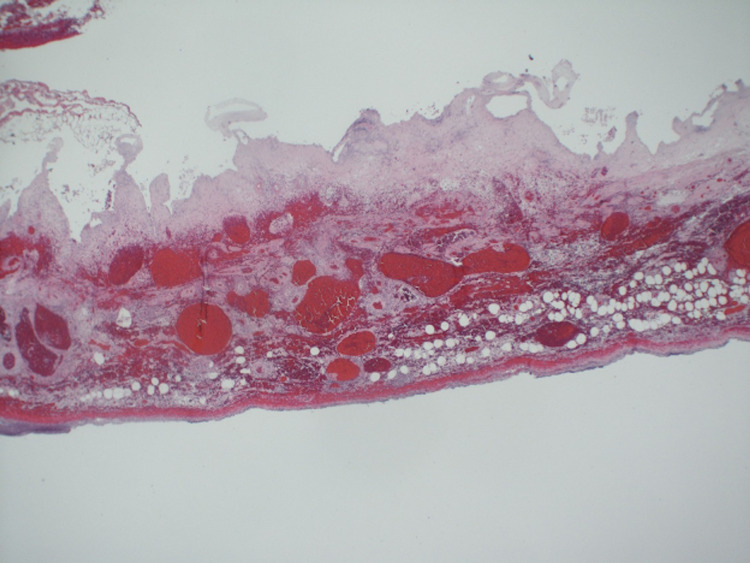
Pathology At lower magnification, the wall of the gallbladder shows transmural necrosis, vascular congestion, hemorrhage, and acute inflammation.

An 86-year-old woman with a history of hypertension, hypothyroidism, hyperlipidemia, colon cancer with sigmoid resection, and lumbar disc disease presented with one day of severe, sharp epigastric pain radiating to the right lower quadrant associated with nausea and vomiting. Physical exam revealed distention, tenderness, guarding, and a palpable mass in the right lower quadrant of the abdomen. There was no rebound tenderness and bowel sounds were hyperactive. Laboratory tests revealed leukocytosis, elevated erythrocyte sedimentation rate, and c-reactive protein. The complete metabolic panel, amylase, lipase, and lactic acid were within normal limits. A CT scan of the abdomen (Figure 1) was completed due to concerns for acute appendicitis or small bowel obstruction; however, imaging revealed the palpable mass on physical exam to be a large cystic ovoid structure in the right anterior abdomen measuring 9.3 x 4.4 x 4.6 cm with no evidence of obstruction or appendicitis. Intravenous contrast was not given, as the patient had elevated creatinine levels and concerns for an acute kidney injury. She was initially treated conservatively with intravenous antibiotics, fluids, anti-emetics, and pain medications. The following day, she had worsening leukocytosis with severe abdominal tenderness and guarding. The decision was made to proceed with emergent laparoscopy as the patient became hemodynamically unstable combined with clinical evidence of peritonitis. Intra-operatively, the gallbladder was noted to be ischemic with necrosis(Figure 2) and was removed. Pathology of the gallbladder (Figure 3) revealed acute hemorrhagic necrosis of the gallbladder without stones confirming the diagnosis of gallbladder volvulus. The patient had an uncomplicated postoperative course and was subsequently discharged two days later on oral antibiotics.

## Discussion

Originally described by Wendel in 1898 [1], gallbladder volvulus is a rare condition, with approximately 400 cases reported in the literature. Many of the documented cases initially described presentations similar to that of acute cholecystitis [2-6]. In 1982, Lau et al. reviewed the clinical features of three cases where a definitive clinical pattern emerged, now defined as the triad of triads [7]. Triad 1 describes patient appearance: elderly, thin body habitus, and chronic chest disease or spinal deformity [5,7]. Triad 2 describes patient symptoms: right upper quadrant pain, sudden and early onset of this pain, and early onset emesis [7]. Finally, Triad 3 describes the physical signs of the patient: palpable right upper quadrant mass, lack of a toxic or jaundiced appearance, and a discrepancy between the pulse and temperature [7]. Serologic testing is non-specific, but as in our patient, typical findings include leukocytosis, elevated inflammatory markers, and normal liver function test. Furthermore, poor response to antibiotic therapy and the absence of cholelithiasis, fever, or jaundice can help differentiate gallbladder volvulus from acute cholecystitis. Although this system was developed to aid in the diagnosis, our case with its abnormal presentation, mimicking acute appendicitis, exhibited four out of nine criteria making the diagnosis more difficult. There are three other documented cases with similar presentations to that of our patient [8-10].

Due to advances in imaging modalities, the incidence of preoperative diagnosis of gallbladder volvulus has increased from 10% to 26% [11]. Because this condition leads to obstruction of biliary drainage and blood flow, a delay in diagnosis and treatment can be life-threatening. There have been only five reported cases of preoperative diagnosis [12].

A pre-operative radiologic diagnosis is challenging, as the actual volvulus may not be visualized. The preferred diagnostic modality is an ultrasound that will commonly demonstrate findings similar to those of acute cholecystitis: a thickened gallbladder wall with pericholecystic fluid [11,13]. Based on her initial presentation, our primary concern was acute appendicitis. Because of this, we decided to obtain a CT scan, which is the preferred diagnostic modality for that condition. Gallbladder wall abnormalities, including the thickening and decreased wall enhancement found in the presence of ischemia, are best seen on a CT scan [8]. A horizontal gallbladder located outside of the gallbladder bed and torsion of the cystic pedicle, known as the “whirl sign,” are also two of the most helpful signs seen on CT scan [3,14]. As noted in Figure 1, our patient was noted to have a horizontal gallbladder but there were no signs of ischemia, wall thickening, or torsion. However, our findings were limited, as the image was completed without intravenous contrast due to the presence of acute kidney injury. Our patient also did not have gallstones, which are present in only 30% of cases of torsion and are not believed to be related to gallbladder volvulus [11,15]. A hydroxy iminodiacetic acid (HIDA) scan shows a “bullseye” image caused by the accumulation of the radioisotope in the gallbladder [8]. Magnetic resonance imaging findings include high signal intensity within the gallbladder wall on T1 weighted images, which suggests necrosis and hemorrhage that may be seen with torsion as well as gangrenous cholecystitis [16]. Magnetic resonance cholangiopancreatography (MRCP) may demonstrate the neck of the gallbladder and cystic duct in more anatomic detail due to the high contrast resolution of this modality [16]. However, a HIDA scan and MRCP were deferred given our patient developed hemodynamic instability with an acute abdomen requiring a surgical approach.

## Conclusions

Despite advances in imaging, volvulus is not usually discovered until surgical intervention, as in our patient. Early diagnosis of gallbladder torsion can help prevent life-threatening complications such as gallbladder gangrene, perforations leading to bilious peritonitis, and other infections. GV should be included in the differential diagnosis of an elderly patient with an acute abdomen. A high index of clinical suspicion is needed in elderly patients, as early diagnosis and definitive surgical intervention reduce morbidity and mortality.
